# Nucleoporin 153 regulates estrogen-dependent nuclear translocation of endothelial nitric oxide synthase and estrogen receptor beta in prostate cancer

**DOI:** 10.18632/oncotarget.25462

**Published:** 2018-06-15

**Authors:** Agnese Re, Claudia Colussi, Simona Nanni, Aurora Aiello, Lorenza Bacci, Claudio Grassi, Alfredo Pontecorvi, Antonella Farsetti

**Affiliations:** ^1^ National Research Council (CNR), Institute of Cell Biology and Neurobiology (IBCN), Rome, Italy; ^2^ Università Cattolica, Institute of Medical Pathology, Rome, Italy; ^3^ Università Cattolica, Institute of Human Physiology, Rome, Italy; ^4^ Fondazione Policlinico Universitario Gemelli, Rome, Italy

**Keywords:** prostate cancer, Nucleoporin 153, eNOS, Estrogen Receptor signaling, molecular biomarkers

## Abstract

Nucleoporin 153 (Nup153), key regulator of nuclear import/export, has been recently associated to oncogenic properties in pancreatic and breast tumour cells modulating either cell motility and migration or gene expression by chromatin association.

In the present work, we have characterized the role of Nup153 in a cellular model of prostate cancer (PCa). The analysis of several immortalized cell lines derived from freshly explants of prostate cancer specimens showed that Nup153 protein was higher and present in multimeric complexes with eNOS and ERβ as compared to normal/hyperplastic prostate epithelial cells. This phenomenon was enhanced in the presence of 17β-estradiol (E_2_, 10^-7^M). Further experiments revealed that eNOS and ERβ were present in a DNA binding complexes associated with Nup153 promoter as demonstrated by ChIPs. Notably, after Nup153 depletion (siNup153), a reduction of migration capacity and colony formation in primary tumor-derived and metastatic PCa cells was observed. In addition, eNOS and ERβ nuclear localization was lost upon siNup 153 regardless of E_2_ treatment, suggesting that Nup153 is a key regulator of prostate cancer cell function and of the nuclear translocation of these proteins in response to hormone stimulus. Taken altogether our findings indicate that in PCa cells: *i.* the expression and function of Nup153 is modulated by estrogen signaling; *ii.* Nup153 contributes to cell migration and proliferation; *iii.* Nup153 regulates the nuclear translocation of eNOS and ERβ by forming a multimeric complex. Our findings unveil Nup153 as a novel component of the estrogen-dependent multimeric complex, thus representing a potential therapeutic candidate in prostate cancer.

## INTRODUCTION

Nucleoporins (NUPs) are components of the nuclear pore complex which spans the nuclear envelope and allows the transport between the cytoplasm and the nucleus. Nucleoporin 153 (Nup153) is placed at the inner part of the nuclear envelope forming the nuclear basket with other peripheral NUPs, where it interacts with the nuclear matrix. Interestingly, several NUPs are capable, upon still unknown signals, to move to nucleoplasm and interact with chromatin, thus contributing not only to nuclear import/export but also to chromatin organization and gene expression regulation. Specifically, it has been recently demonstrated that Nup153 represents a new class of global chromatin-binding proteins regulating the spatial organization of chromosomes associating at very high density with transcriptionally active regions [1, 2]. NUPs, including Nup153, appear to be implicated in a large number of disorders, such as cardiomyopathy associated to muscular dystrophy [2], autoimmune disease and cancer [3, 4]. In this regard, several reports established that NUPs regulate cancer through different mechanisms based either on the import of DNA repair proteins [5] or gene regulation [3]. Increased expression of Nup153 due to a 6p22 genomic translocation was detected in urothelial carcinoma and retinoblastoma [6, 7]. Moreover, in a screening for genes, Nup153 was found amplified in the pancreatic cell line PL5 [8]. This study suggested an oncogenic function for Nup153 by modulating the TGF-β signalling pathway. Furthermore, Nup153 is important for tumor cell migration and proliferation [9] which are essential features of metastatic cancers. Of note, Nup153 knockdown was responsible for the alteration of nuclear lamin A in these cancer cells. Indeed, the function of nuclear envelope and nuclear matrix are intimately connected and linked to chromatin structure and integrity and are often altered in cancer cells.

Previous works from others and our group identified a molecular mechanism that sustains the aggressive phenotype of prostate cancer (PCa) [10, 11]. The endothelial nitric oxide (NO) synthase (eNOS) in fact, plays a key role in prostate tumor maintenance and progression by forming complexes with Estrogen Receptors (ERs) [12–14] which exert an epigenetic control of gene expression. Importantly, the localization of eNOS-containing nuclear complexes, as assessed by ChIP-Seq, shows a specific pattern in response to 17β-estradiol (E_2_) treatment contributing to the acquisition of a pathological gene expression signature. Overall, these findings are in agreement with the concept that NO has a broad negative effects on cancer, sustaining cell malignant transformation, cancer progression and the metastatic cascade ([15] and references therein).

Here we report about the new role of Nup153 in a hormone-driven activation cascade, involving eNOS and NO production, which contributes to a transcriptional program associated with prostate cancer. In fact, hormone-dependent eNOS activation enhances Nup153 expression which is followed by nuclear import of eNOS/ERβ by the formation of eNOS/ERβ/Nup153 complexes. Our results suggest Nup153 as potential therapeutic target in prostate cancer.

## RESULTS

### Nup153 regulates cell migration and colony formation in prostate cancer cells

To investigate Nup153 potential contribution to the biology of prostate cancer two cellular models were adopted: human immortalized cell lines derived from freshly explanted prostate tumors obtained from patients with diagnosis of clinically localized disease and selected for the absence of hormonal neoadjuvant treatment before surgery [16] and the metastatic prostate cancer cell line LNCaP. We first evaluated whether Nup153 silencing by siRNA could affect migration and clonogenic ability of prostate cancer cells.

**Figure 1:**
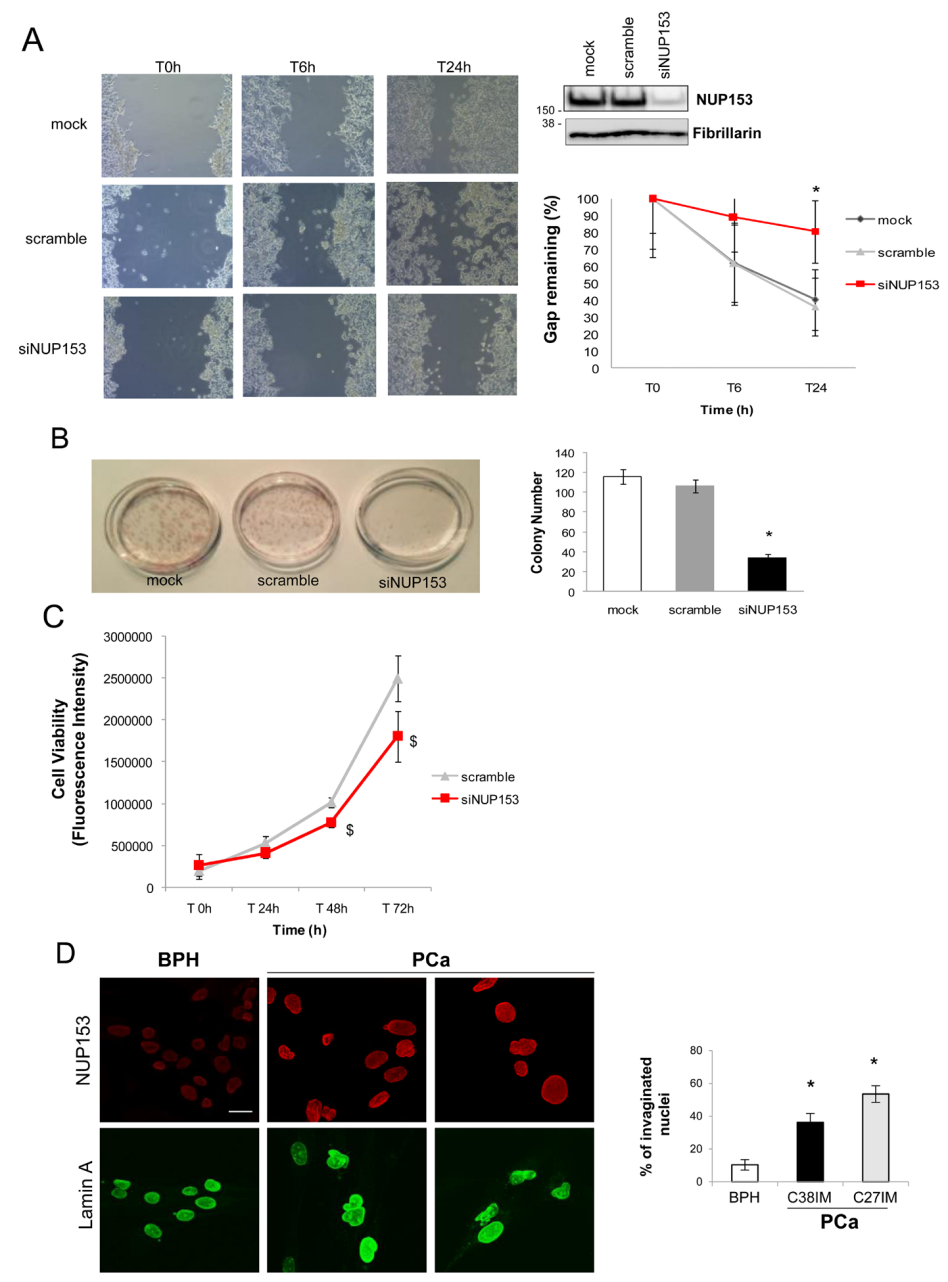
Nup153 depletion in PCa cells regulates migration and colony formation **(A)** Representative phase contrast images (Bright field) of a scratch test in a time course of 24h (left panel) and its quantification (right panel, down), expressed as percentage of remaining gap, in Nup153 silenced (siNUP153 oligos mix; 60nM), scramble control and mock transfected LNCaP cells. The wound was made 48h after transfection. Nup153 protein level in the above conditions is shown in the western blot (right panel, up). **(B)** Left panel: Representative digital image showing colonies produced by PCa (C27IM) cells after plating 150 cells. Cells were transfected in the same experimental conditions of scratch test and, after eight days, were fixed and stained with hematoxilin. In the right panel, the number of colonies for each condition is shown. Data expressed as mean +/-SEM of 3 independent experiments. ^*^ p< 0.05 vs mock. **(C)** Cell Viability in a time course of 72h in the same experimental conditions as described for scratch test. Data are expressed as mean +/-SEM of 3 independent experiments in triplicate, corrected to fluorescence background. ^$^ p< 0.05 vs scramble. **(D)** Confocal analysis of PCa (C27IM and C38IM) and Benign Prostatic Hyperplasia (BPH, C17IM) cells stained with antibodies to Nup153 ([QE5]; red) or Lamin A (green). Scale bar: 20 μm (Nup153 and Lamin A). The graph shows the percentage of nuclei bearing invaginations (right panel). ^*^ p< 0.05 vs BPH.

A significant reduction of migration capacity as well proliferation was found in primary tumor-derived (C27IM, not shown) and metastatic (LNCaP) PCa cells undergoing to Nup153 downregulation as shown by scratch test and colony formation assay (Figure 1A-1B). Nup153 silencing progressively reduced cell metabolic activity (Figure 1C), in agreement with its essential role in cell viability and control of cell cycle [17–19].

When we analysed Nup153 expression by confocal microscopy we found that in the unstimulated conditions Nup153 protein level was higher in PCa cells (C38IM and C27IM) as compared to Benign Prostate Hyperplasia (BPH) cells (C17IM) (Figure 1D, upper panels). Nup153 knockdown has been previously linked to alteration of the nuclear lamin A in breast cancer cells [9]. Interestingly, in PCa cells (C38IM and C27IM), that show increased level of Nup153, the organization of nuclear lamin A, was also altered and the nuclei showed irregular shapes with membrane invaginations and multiple lobes (Figure 1D, lower panels and right graph).

### Hormone-dependent eNOS activation regulates Nup153 expression in PCa cells

**Figure 2:**
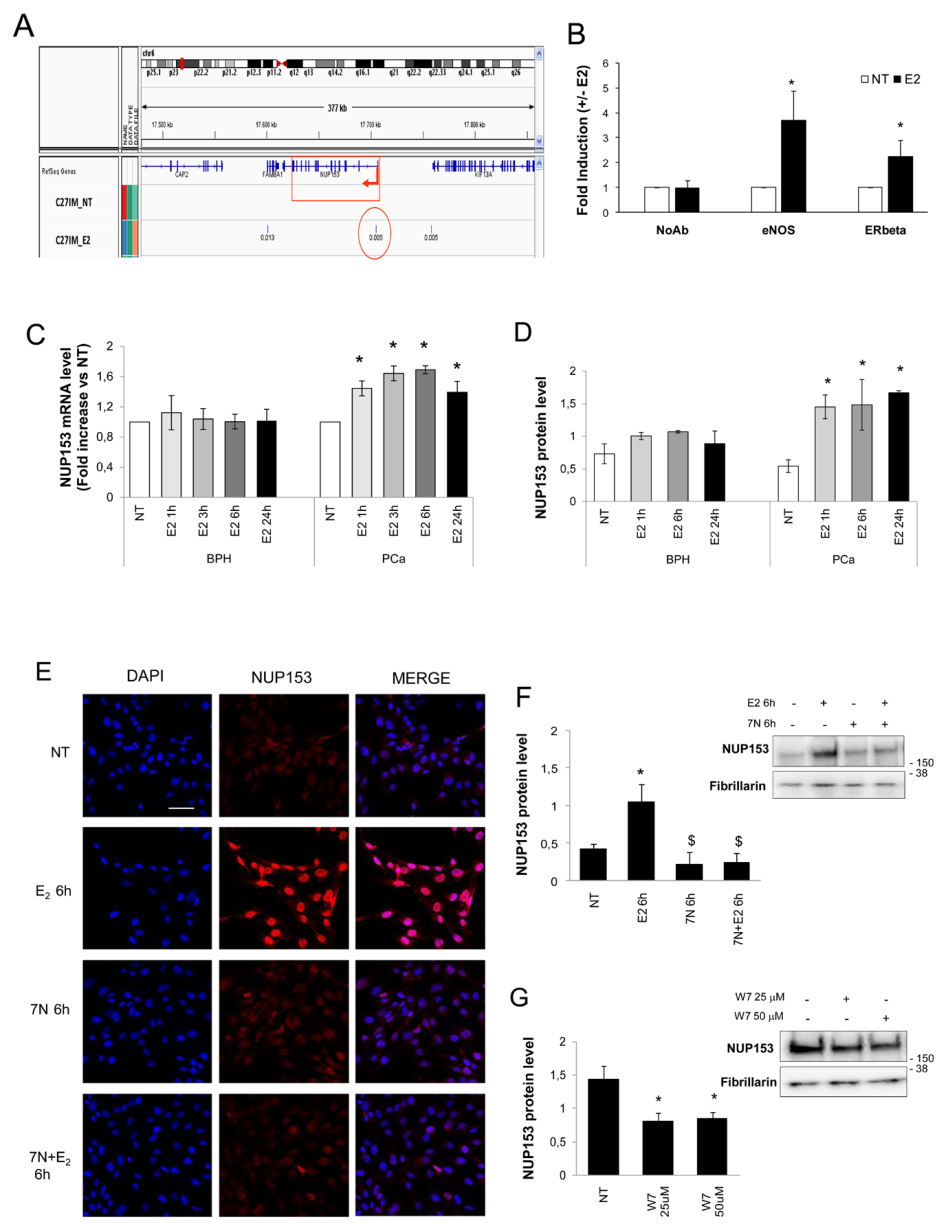
eNOS binds Nup153 promoter and regulates its expression in an estrogen-dependent manner **(A)** Integrated Genome Viewer (IGV 2.3) screenshot showing peaks of eNOS identified by ChIP–Seq at the genomic regions encoding Nup153 in PCa cells (C27IM) in the absence (NT) or presence of 17β-estradiol (E_2_, 10^-7^M, 45min). Region amplified in panel B is indicated with a red circle. **(B)** Recruitment of eNOS and ERβ on the promoter region of Nup153 by ChIPs in the presence or absence of E_2_ (45 min) in C27IM cells. The immunoprecipitations were performed using anti-eNOS (Type III, BD), anti-ERβ (L-20) or no antibody (NoAb) as a negative control. Values are represented as fold of induction (+/-E_2_) and as mean +/-SEM of 3 independent experiments. ^*^p<0.05 E_2_ vs NT. **(C)** Nup153 mRNA levels quantified by *q*RT-PCR in PCa and BPH cells in basal condition (NT) and after E_2_ in a time course of 1, 3, 6 or 24 hours. The results are plotted as fold induction vs NT +/-SEM of 3 independent experiments.^*^p<0.05 E_2_ vs NT. **(D)** Nup153 protein levels were analysed by western blot in PCa and BPH cells in a time course of 1, 6 or 24 hours. Numbers represent optical density analysis of bands normalized to loading control. Data are mean +/-SEM of 3 and 4 independent experiments for BPH and PCa, respectively. ^*^p< 0.05 vs NT. **(E)** Confocal analysis of Nup153 ([QE5]; red) in PCa cells untreated (NT) or treated with E_2_ (10^-7^M) and/or 7N (0.5 mM) for 6 hours. Nuclei were counterstained with DAPI (blue) and merged with the Nup153 signal (red). Scale bar 25μm. **(F-G)** Protein level analysis of Nup153 before and after E_2_ (10^-7^M, 6h) alone or in combination with 7N (0.5 mM, 6h) (F) and upon W7 (G) treatment (25 μM and 50μM, overnight). Right Panel: Representative western blot of Nup153. Fibrillarin served as loading control. Left Panel: Numbers represent optical density analysis of bands normalized to loading control. Data are mean +/-SEM of 3 for W7 and 4 for 7N independent experiments. ^*^p< 0.05 vs NT; ^$^ p< 0.05 vs E_2_.

Our PCa cell lines show a particular aggressive phenotype that we found to be mainly dependent on a basal higher estrogenic responsiveness that determines in turns eNOS and ERβ nuclear accumulation and complexes formation, which drive a pathological gene expression [13, 14]. Thus, we reasoned that the higher basal level of Nup153 in PCa cells compared to BPH could be a consequence of a potential hormone-responsiveness of Nup153, mediated by eNOS activation, in prostate microenvironment [12–14, 20–24]. In order to understand the contribution of estrogenic signalling in this pathway, PCa cells were cultured for 72 hours in hormone-deprived serum and then treated or not with 17β-estradiol. In the above conditions, we analysed the regulatory region at 5’ Nup153 gene on our ChIP-Seq database [12]. Intriguingly this analysis revealed a hormone-dependent localization of eNOS-DNA association peaks along Nup153 extragenic/intronic regions, strongly supporting the hypothesis of an estrogen-dependent regulation of Nup153 (Figure 2A). By an independent set of traditional ChIP in C27IM cells we validated the hormone-induced eNOS binding downstream to TSS within Nup153 genomic region, confirming that Nup153 is a transcriptional target of eNOS. In addition, an estrogen-dependent recruitment of ERβ was also found onto Nup153 promoter, suggesting the formation of a combinatorial chromatin complex eNOS/ERβ along that genomic region (Figure 2B). To further corroborate our finding a second antibody to ERβ, the CWK-F12 [25, 26], also suitable for ChIP assays, was used, confirming recruitment of ERβ in basal as well in the estrogen-stimulated condition onto Nup153 promoter (Supplementary Figure 1).

We next investigated the responsiveness to E_2_ of Nup153 in terms of RNA and protein expression. To address this point, *quantitative* RealTime PCR (*q*RT-PCR), western blot, and confocal microscopy were performed in PCa and BPH cells cultured for 72 hours in hormone-deprived serum and then treated with 17β-estradiol (E_2_;10^-7^M) for 1, 3, 6 and 24 hours. In BPH cells Nup153 mRNA level was weakly affected by E_2_ treatment exhibiting a similar extent at each time course considered (Figure 2C), while an induction of Nup153 was observed in PCa cells. Similar results were obtained for Nup153 protein expression that was unaffected in BPH and induced in PCa cells by E_2_ treatment (Figure 2D). Hormone-driven Nup153 protein increase was prevented in the presence of 7-nitroindazole (7N) an inhibitor of the NOS function as demonstrated by confocal and western blot analyses (Figure 2E–2F). The key role of eNOS in regulation of Nup153 expression was confirmed using W7, an inhibitor of Calmodulin function, which is upstream of the eNOS activation, determining Nup153 downregulation in PCa cells (Figure 2G).

### Nup153 is part of a multimeric complex induced upon estrogen stimulation

**Figure 3:**
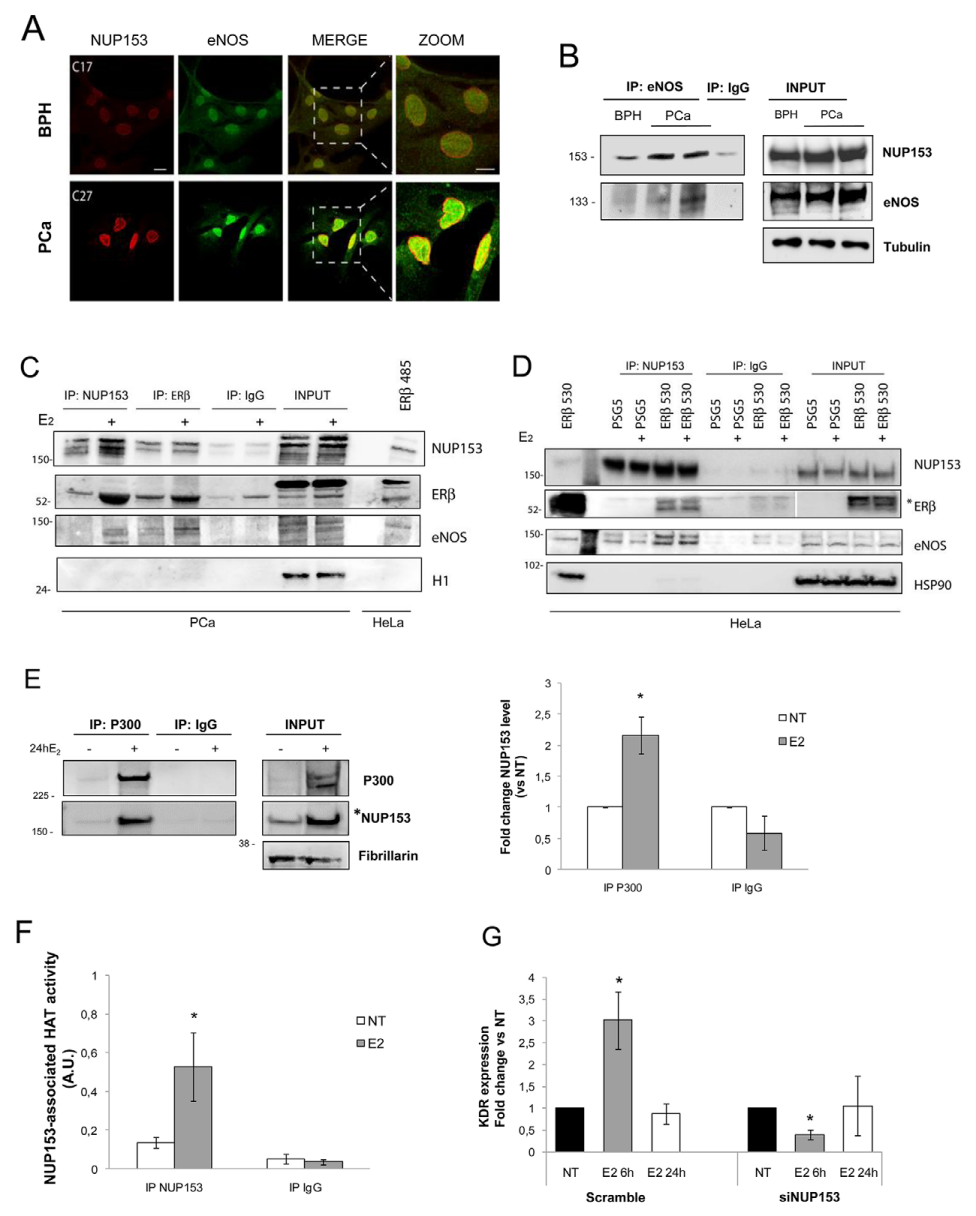
Nup153 forms complexes with eNOS, ERβ and p300 **(A)** Confocal analysis of Prostate Cancer (PCa, C27IM) and Benign Prostatic Hyperplasia (BPH, C17IM) cells stained with antibodies to Nup153 ([QE5]; red) or eNOS (Type III; green). Scale bar: 20μm (Nup153, eNOS and MERGE; zoomed area is showed). **(B)** Analysis of Nup153 interaction with eNOS by Co-Immunoprecipitation in PCa and BPH cells in basal condition. Immunoprecipitation with IgG served as negative control. **(C)** Analysis of Nup153 interaction with eNOS and ERβ by Co-Immunoprecipitation in PCa cells in basal condition or upon E_2_ treatment (10^-7^M, 24h). PCa cells were immunoprecipitated with Nup153 Antibody ([7AB], Abcam) or with ERβ (GeneTex, #110607). Immunoprecipitation with IgG served as negative control. Membranes were blotted with specific antibodies to Nup153 ([Q5], Abcam), ERβ (GeneTex, #110607) and eNOS (Type III, BD). H1 served as control. 4μg of transfected HeLa with human ERβ short isoform (485aa) were used as positive control for Estrogen Receptor. Total proteins were resolved by SDS-PAGE using a 10% Invitrogen precast gel (NuPage and MES buffer). **(D)** Analysis of Nup153 interaction with eNOS and ERβ by Co-Immunoprecipitation in HeLa cells transfected with empty vector (PSG5) or with human ERβ full-length (ERβ 530aa), before and after E_2_ treatment (10^-7^M, 24h). HeLa cells were immunoprecipitated with Nup153 Antibody ([7AB], Abcam). Immunoprecipitation with IgG served as negative control. Membranes were blotted with specific antibodies to Nup153 ([QE5], Abcam), ERβ (CWK-F12, DHSB) and eNOS (Type III, BD). Hsp90 served as control. Total cell lysates (15μg) from HeLa transfected with human ERβ 530aa isoform were used as positive control for Estrogen Receptor. ^*^ indicates a lower exposure for INPUT and positive control, respectively. Total proteins were resolved by SDS-PAGE using a 3-8% Invitrogen precast gel (NuPage and TA buffer). **(E)** Analysis of Nup153 interaction with Histone Acetyl Transferase p300 by co-immunoprecipitation in PCa cells in basal condition or upon E_2_ treatment (10^-7^M, 24h). Immunoprecipitation with IgG was used as negative control. Fibrillarin served as loading control. ^*^ indicates a lower exposure for INPUT. Densitometric analysis showing, in the right panel, the interaction of Nup153 with p300 in E_2_ treated samples versus NT, normalized to input loading control and expressed as fold change. Data are mean +/-SEM of 3 independent experiments. ^*^p< 0.05 vs NT. **(F)** Evaluation of HAT activity specifically associated with Nup153 in total extracts of PCa cells cultured in absence or in presence of E_2_ (10^-7^M, 24h) and subjected to immunoprecipitation with anti-Nup153 antibody ([7AB], Abcam) or normal IgG immunoglobulin as negative control. Data are expressed as mean +/-SEM of 3 independent experiments. A.U. Arbitrary Unit ^*^p<0.05 E_2_ vs NT. **(G)** Effect of Nup153 depletion on estrogen response gene. VEGF Type2 Receptor (KDR) mRNA levels quantified by *q*RT-PCR in PCa cells before (scramble) and after Nup153 depletion (siNUP153 oligos mix, 60 nM), untreated or treated with E_2_ (10^-7^M, 6 and/or 24 hours). The results are plotted as Fold change vs NT, +/-SEM of 5 independent experiments.^*^p<0.05 E_2_ vs NT.

We previously demonstrated that eNOS nuclear import, followed by the formation of transcriptional complexes with ERβ, occurs in PCa cells under basal condition and is enhanced under estrogen stimulus [13]. To understand whether and how Nup153 may take part of this activation pathway we investigated the possible formation of protein complexes potentially important for both eNOS nuclear translocation and its activation for transcriptional activity. We analysed the cells either in basal condition or after estrogenic deprivation (NT) followed or not by E_2_ treatment for 3h and 45min and/or 24h (E_2_). Confocal microscopy analysis revealed co-localization of eNOS and Nup153 predominantly in PCa cells in basal condition (Figure 3A). Quantification of double-labelled areas in BPH or PCa cells was 2% *vs*. 38%, respectively (*not shown*). Co-IP for eNOS (Figure 3B) confirmed an interaction between Nup153 and eNOS that was stronger in tumor (PCa, C38IM and C27IM) as compared to BPH (C17IM) cells used as normal control.

Additional Co-IP experiments were performed in PCa cells (C27IM), endogenously expressing ERβ, before and after E_2_ stimulation. Either immunoprecipitation for ERβ and Nup153, revealed that hormone treatment induced the formation of a trimeric complex containing eNOS, ERβ and Nup153 (Figure 3C). To reinforce our finding we used an alternative antibody proved highly specific for ERβ and an additional cell line, HeLa, known to be ER-negative. We performed a Co-IP for Nup153 (Figure 3D) in HeLa transfected with human ERβ full-length in basal or upon E_2_ treatment. Results confirmed a specific interaction among Nup153, ERβ and eNOS in HeLa cells also using this different antibody for ERβ.

Nup153 association with histone acetylases (HATs) regulates their activity in cardiomyocytes bearing dystrophin mutation [2]. Other reports also have shown that epigenetic enzymes function and chromatin association are regulated by nucleoporins [27]. Thus, we first investigated by Co-IP the potential Nup153 interaction with the HAT member p300 and found that E_2_ treatment strongly enhanced their association in PCa cells compared to un-stimulated and estrogen-deprived cancer cells (Figure 3E). We found also, that E_2_ was capable of modulating the acetylase activity specifically associated with Nup153 (Figure 3F) suggesting the formation of a transcriptionally competent complex upon estrogen stimulation.

To explore further the role of Nup153 in the E_2_ signalling we analysed the expression of VEGF Type2 Receptor (VEGFR2/KDR), an E_2_-target gene, after Nup153 silencing and under hormone stimulation. Nup153 depletion virtually abrogated VEGFR2/KDR E_2_-responsiveness thus confirming Nup153 transcriptional role in this context (Figure 3G).

### Nup153 regulates eNOS and ERβ nuclear import in a hormone-dependent manner

The evidence of the nuclear interaction between eNOS and ERβ with Nup153 prompted us to investigate whether Nup153 had a role in nuclear transport of these proteins. By confocal microscopy, we analysed eNOS and ERβ nuclear shuttling, upon E_2_ treatment, in PCa cells that were silenced for Nup153 or treated with scramble oligo as control.

**Figure 4:**
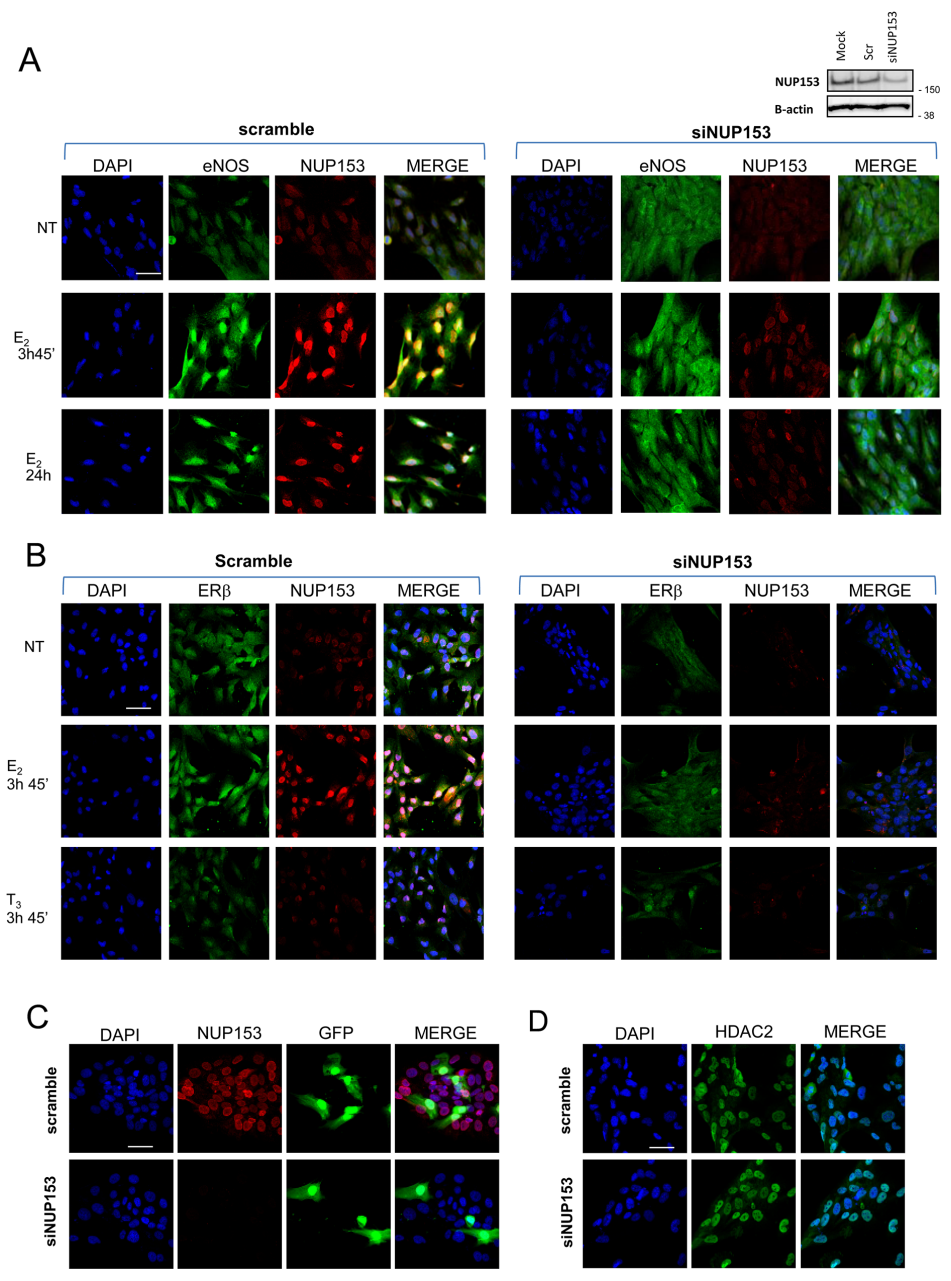
Nup153 controls eNOS and ERβ nuclear translocation **(A-B)** Representative confocal microscopy images of eNOS (A) or ERβ (B) nuclear localization (green) in PCa cells before and after Nup153 depletion, untreated or treated with E_2_ (10^-7^M, 3h45min and/or 24 hours). Cells silenced for Nup153 (oligos mix, 60nM) or treated with scramble control oligo were analysed at 48h post-transfection. T_3_ treatment (10^-7^M, 3h45min) served as control of ERβ estrogen response specificity. Nuclei were counterstained with DAPI (blue) and merged with the Nup153 ([QE5], Abcam; red) and eNOS (Type III, BD; green) or ERβ (Genetex #110607; green) signals. Efficiency of Nup153 interference is assessed also by western blot (right upper panel A). **(C)** Representative confocal microscopy images of GFP localization in PCa-GFP transfected cells before and after Nup153 depletion. Nuclei were counterstained with DAPI and merged with the two protein signals (GFP in green and Nup153 [QE5] in red). Scale bar 50 μm. **(D)** Representative confocal microscopy images of HDAC2 localization (green) in PCa cells before and after Nup153 depletion. Nuclei were counterstained with DAPI and merged with the HDAC2 signal (green). Scale bar 50 μm.

Nup153 expression was highly induced upon E_2_ treatment at the two time points (3h45min; 24h) and in parallel eNOS and ERβ were imported into the nucleus (Figure 4A–4B). However, Nup153 silencing strongly reduced the presence in the nucleus of eNOS and ERβ both in NT and in E_2_ treated cells (Figure 4A–4B). Similar results were obtained in an additional immunofluorescence experiment performed with the validated antibody to ERβ (CWK-F12, DHSB) and a rabbit antibody to Nup153 ([H-161], Santa Cruz) (Supplementary Figure 2).

To assess the specificity of Nup153-dependent eNOS and ERβ nuclear import we evaluated the effects of Nup153 depletion on nucleo-cytoplasmic protein trafficking, with particular attention to the localization of the exogenous Green Fluorescent Protein (GFP), in p-GFP transfected cells, and the endogenous Histone deacetylase 2 (HDAC2) proteins. No modification of GFP and HDAC2 localization was indeed observed upon siNup153, thus indicating that inhibition of eNOS and ERβ nuclear translocation after Nup153 depletion is specific (Figure 4C–4D).

## DISCUSSION

Many studies demonstrated a link between aberrant nuclear shape and tumorigenesis. Indeed, components of the nuclear envelope have been implicated in the regulation of several cell functions affected during cancer initiation and progression, such as proliferation, migration and DNA repair. In this context we have found that the nuclear pore component Nup153 plays a key role in the aberrant estrogen response of prostate cancer, promoting the nuclear import of important determinants of transcription such as ERβ and eNOS which have been demonstrated to cooperate in driving the pathological gene signature.

Moreover, under estrogen induction, these effectors bind to Nup153 promoter region, increasing its protein level and consequently the nuclear import of eNOS/ERβ thus amplifying the effects on gene expression alteration and contributing to tumor aggressiveness and the negative prognosis for PCa.

The metastatic form of prostate cancer, in fact, is generally refractory to therapy and leads rapidly to death. Although the mechanistic control of prostate cancer cell metastasis is still poorly characterized, it is recognized as a multi-step process in which the epithelial-mesenchymal transition and the acquisition of enhanced cell motility largely contribute to the acquisition of the metastatic phenotype.

Our results showing that Nup153 positively regulates cell migration and is associated with aberrant nuclear structure in PCa is in line with previous findings describing the role of this nucleoporin in nuclear shaping and cell motility in other cancers [9].

Nucleoskeleton and cytoskeleton are intimately connected through the LINC complex [28] thus changes in nuclear structural proteins such as Nup153 and Lamins, may directly affect cell migration increasing the nuclear plasticity necessary for cell deformation during intra-extravasation process that characterizes metastatic cells [3]. Another important finding of our work regards the positive effect of Nup153 on the clonogenic capacity of prostate cancer cells thus suggesting that targeting this protein could represent a possible therapeutic strategy to counteract tumor growth. In fact, NUPs are important regulators of cell cycle either by taking part in the assembly-disassembly process during mitosis or by export of cell cycle inhibitors [29].

In the present study, we found that Nup153 is overexpressed in primary and metastatic PCa tumor cells as compared to benign lesions. Chromatin immunoprecipitation analysis suggests that a complex encompassing eNOS and ERβ is present on Nup153 promoter region and may then regulate its transcription as previously reported for other genes belonging to a prognostic signature including the telomerase catalytic subunit [13].

In prostate cancer the estrogen responsiveness, high levels of ERβ expression [30] associated with nuclear accumulation of eNOS [13] may represent a negative prognostic condition. Although the signal triggering Nup153 expression is still unclear we observed that Nup153 expression was induced by estrogens and associated with the estrogen property to upregulate the intracellular synthesis of nitric oxide (NO). Inhibitors affecting NO synthesis or eNOS activation pathway, in fact, abrogated the estrogen-dependent Nup153 protein increase. This observation is of particular interest indicating that both estrogens and NO may cooperate and contribute to aggressiveness in prostate cancer [13] through Nup153 upregulation.

Prior work described that Nup153 is involved in epigenetic processes aimed at controlling nuclear trafficking and gene expression [31]. Intriguingly, Nup153 has be found associated with chromatin in different cellular contexts possibly facilitating expression of a number of target genes via microRNAs and small interfering RNAs [1, 32]. In this light, the association of Nup153 with both ERβ and eNOS in a trimeric complex not only is required for nuclear co-translocation of these proteins, but also could function as epigenetically active scaffold allowing their activity.

**Figure 5:**
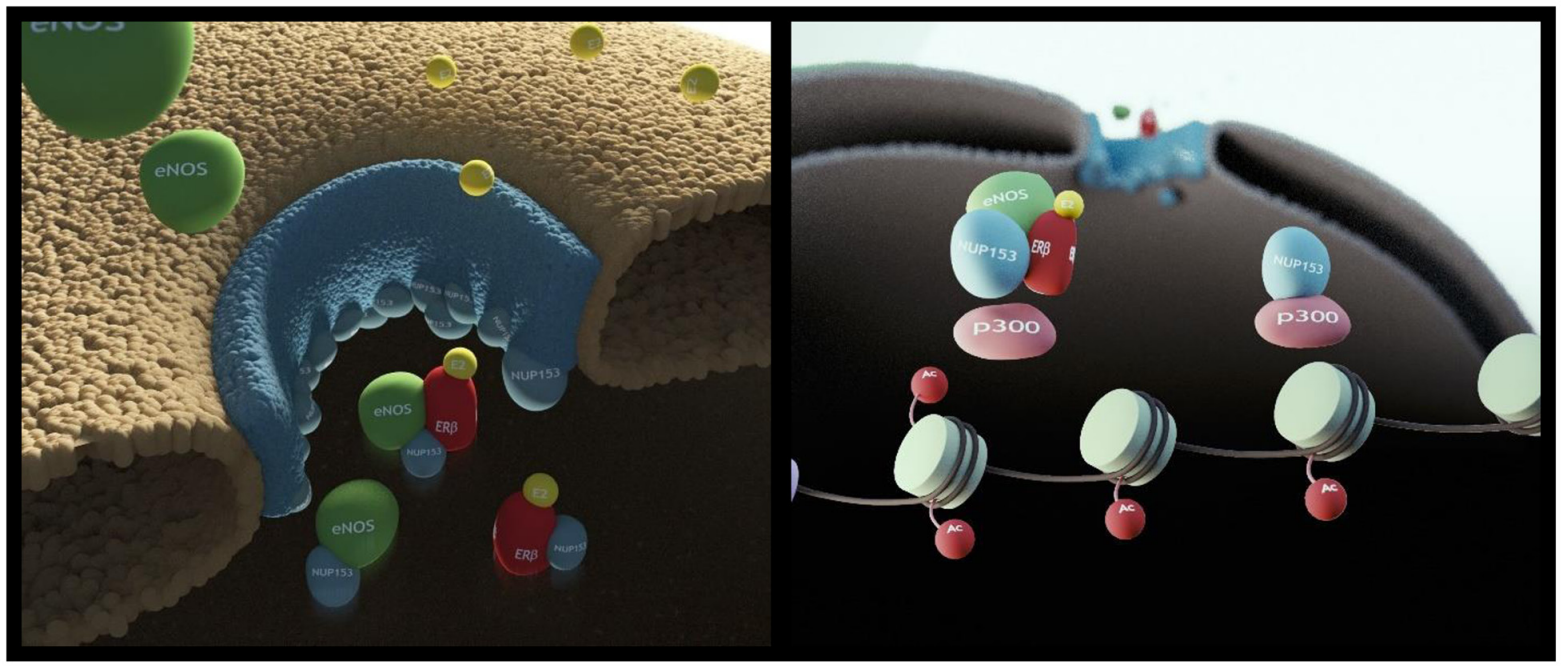
Cartoon depicting the proposed role for Nup153 as regulator of eNOS and ERβ nuclear translocation and as scaffold for p300 Specifically, Nup153 binds eNOS and ERβ and drives their nuclear translocation. Once in the nucleoplasm Nup153 interacts with p300. Estrogen signaling affects Nup153 function by favouring its association with eNOS, ERβ and p300 and by increasing the acetylase activity specifically associated with Nup153.

The association of Nup153 with other epigenetically active components has been previously reported in different conditions. Specifically, nucleoporins association with HDACs has been shown important for their proper chromatin localization and gene regulation [27, 33, 34]. Prior studies, however, indicated that Nup153 could be acetylated in the heart of dystrophic mice. In this context, it has been found associated with lysine acetylases including p300 and PCAF [2]. These epigenetic enzymes function as transcription co-activators facilitating the expression of genes present in the chromatin region to which they are associated. Remarkably, lysine acetylases are always present as cofactors in nuclear hormone receptor complexes including those in which ERβ is present [35]. In our study, it also emerged that p300 was associated with Nup153 in prostate cancer cells. This experimental evidence together with the specific Nup153-associated histone acetylase activity significantly incremented in the presence of estradiol is suggestive of a functional regulatory loop determining an estrogen-dependent Nup153 protein complex formation (see cartoon in Figure 5). In conclusion, the present report highlights the role of the nuclear envelope environment as a new player in prostate cancer cell biology. In response to estrogens and nitric oxide, Nup153 in fact, regulates ERβ and eNOS function through a coordinated nuclear import and providing an epigenetic scaffold for complex formation and appropriate release of the transcriptionally competent complexes in the nucleoplasm. The pathological gene expression that is estrogen-dependent in PCa is a major determinant of bad prognosis and of refractory and metastatic tumors. In this light, although further studies are required, our findings may suggest that, in addition to standard interventions, targeting Nup153 could be a potential new strategy. In fact, Nup153 depletion or inactivation could block the pathological estrogen signaling that sustains the aberrant gene expression in prostate cancer cells thus helping to normalize their epigenetic profile, to reduce proliferation and migration with important effects on their aggressive phenotype and cancer recurrence. This concept is indeed substantiated by our finding of abrogation of the VEGFR2/KDR estrogen responsiveness upon siNup153, given the relevance of the VEGF-VEGFR2 signaling in tumor-associated angiogenesis. Thus, in conclusion, the multiple roles played by Nup153 in prostate cancer cells makes it a potential powerful target to counteract tumor aggressiveness.

## MATERIALS AND METHODS

### Antibodies

The following antibodies were used: anti-ERβ[L-20] (#sc-6822), anti-p300 [C-20] (#292438), anti-Nup153[H161] (#sc-292438) from Santa Cruz Biotechnology, Dallas TX, USA; anti-ERβ from GeneTex (#110607), Irvine, CA, USA and CWK-F12 from DSHB, Iowa City, IA, USA; anti-eNOS Type III (#610299) from BD Biosciences, Franklin Lakes, NJ, USA; anti-Nup153 [QE5] (#Ab24700) for WB and confocal microscopy, and [7A8] (#Ab93310) for Co-IP from Abcam; anti-Fibrillarin [38F3] from Pierce (#MA3-16771); anti-Lamin A [131C3] (#Ab8984) from Abcam; anti-tubulin from Immunological Science (#MAB-10285); anti-HSP90 (#SPC-104C)from StressMarq Biosciences Inc; anti-mouse FITC (#715095150) and anti-rabbit TRITC (#711025152) from Jackson Immunological Research.

### Cell culture and treatments

PCa (C27IM, C11IM and C38IM), LNCaP and HeLa cell cultures and treatments were as in [12–14, 36]. All media were supplemented with 10% fetal bovine serum, 2 mM glutamine, 100 ug/ml penicillin and streptomycin. At least 72 h before experimental use, cells were switched to a medium with hormone-deprived serum and treated with 17β-estradiol (E_2_), triiodothyronine (T_3_), N-(6-Aminohexyl)-5-chloro-1-naphthalenesulfonamide hydrochloride (W7) or 7-nitroindazole (7N), for the concentrations and the times indicated in the figure legends.

### Scratch test

Scratch assay was performed as described in [37]. Briefly, cells were transfected and cultured to confluence. The monolayer was scraped with a p200 pipet tip in a straight line to create a “scratch”. Images were obtained by using a phase-contrast microscope (AXIO microscope with AxioCam ERc5s, Zeiss- objective 10X) in a time course of 0, 6 and 24 hours, distance between edges was measured by the phase contrast microscopy and analyzed by ZEN imaging software (Zeiss). Data were represented as percentage of residual gap.

### Colony formation assay

Colony formation assay was performed as described previously [38]. Briefly, about 2×10^2^ cells were plated into a 6-well culture. After incubation at 37°C for 8 days, the cells were washed twice with PBS, fixed in methanol and stained with Hematoxylin. The number of colonies were counted under microscope. Each sample was run in triplicate.

### Cell viability assay

Cell Viability Assay was performed according to manufacturer's instructions of The CellTiter-Blue Cell Viability Assay (Promega). Briefly, about 2×10^3^ cells untreated (mock) and transfected with scramble oligo or siNup153 oligos mix (60nM) in Reverse mode were added into a 96-well culture plate with three replicates for each group. The cells were incubated for 24, 48 and 72 h. Before the test, cells were washed (with PBS, three times) and fresh medium (100 μL) containing the CellTiter-Blue (20 μL) was added. The plate was incubated for 1-4 h, protected from light at 37°C with 5% CO_2_, before the measurement of the fluorescent signal. Background fluorescence was corrected by including control wells on each plate/time tested (24h, 48h and 72h) to measure the fluorescence from serum-supplemented culture medium in the absence of cells.

### HAT activity

Measurement of the lysine acetylase (HAT) activity was analyzed as previously described [2, 39]. Briefly, Nup153-associated HAT activity was evaluated after immunoprecipitation using 400 μg of protein extract. Normal IgG served as negative control. 40μg of protein extract was used to evaluate total HAT (BioVision) activity following manufacturer's instructions.

### Confocal microscopy

Confocal analysis was performed as previously described [2, 12–14].

Related to Supplementary Figure 2 the cells were fixed with methanol/acetone (1:1) for 10 min at 20°C and then permeabilized for 5min in 0.2% Triton-X100 in PBS. After a brief rinse non-specific sites were blocked with BSA 5%, NGS 3% in PBS for 1h. The following antibodies were used for an overnight incubation: anti-ERβ (CWK-F12, 1:250, monoclonal), anti-Nup153 ([H-161], Santa Cruz #sc-292438, 1:100, polyclonal). The following day, FITC and TRITC secondary anti-mouse and anti-rabbit antibodies (1:500, Jackson Immunological Research) were incubated for 1h. After washes nuclei were stained with DAPI.

Samples were analyzed with a confocal laser scanning system (TCS-SP2; Leica Microsystems and/or Nikon-Ti Eclipse equipped with a 20x-40x objective). Co-localization of the two proteins was performed analyzing z-stacked images. Confocal settings were the same for all examined samples in order to compare fluorescence intensities.

### Western-blot assay and co-immunoprecipitation

Protein extracts were obtained as in [2, 12, 40]. Specifically, samples were lysed in buffer containing 50mM Tris-HCl (pH 7.4), 250 mM NaCl, 0.1% tritonX100, 5mM EDTA, 0.3% Empigen BB and supplemented with 1mM PMSF and protease inhibitor mix. Western Blot assay was performed using 40 μg of total extract. Co-immunoprecipitation experiments were performed using 4 μg of antibody for a range of 500-700 μg of protein extract. Ademtech's Bio-Adembeads paramagnetic bead system was used to immunoprecipitate the specific proteins. A negative control was performed with the same amount of protein extract sample immunoprecipitated with the corresponding purified IgG (Santa Cruz). Total proteins were resolved by SDS-PAGE using a 3-8% gradient and/or 10% Invitrogen Precast gel (NuPage, TA or MES buffer). Specific protein signals were revealed with ECL Prime (Amersham, GE Healthcare) and detected by UVIDOC (Eppendorf S.r.l.). The intensity of each band was evaluated by using UVIDOC and/or the NIH Image J 1.8 software (National Institutes of Health, Bethesda, Maryland, USA). Optical density values of specific proteins were normalized to that of tubulin, H1, HSP90 or fibrillarin.

### RNA interference and transfection

Small interference to Nup153 was obtained with 60nM Trilencer-27mer siRNA duplexes as oligos mix (Origene, Rockville, MD, USA) transfected by Lipofectamine RNAiMAX according to the manufacturer's instructions in hormone-deprived serum and after 48h treated with 17β-estradiol (E_2_). Overexpression of ERβ was obtained with Expression vectors encoding the human ERβ full-length (530aa) or short isoform (485aa) as described [13].

### RNA extraction and qRT analysis

RNA isolation, cDNA preparation and RNA quantification were performed as described [12, 13]. Briefly, real-time PCR (*q*RT-PCR) was repeated three times in duplicate on an ABI Prism 7500 Detection System and/or QuantStudio 5 Real-Time PCR System (Applied Biosystems). Aldolase and Gapdh served as endogenous controls.

Primers sequences, used with SYBR Green Master Mix (Applied Biosystems), were as described in [13] and as follow:

hNUP153:

5’-TGATAACATCTCAACTACCAGTGGTTT-3’ and 5’-GAGAGTGAGTGAGAACGTTCAGCTT-3’.

### Chromatin immunoprecipitation

Chromatin cross-linking and immunoprecipitation (ChIP) assays were performed as described [13], using antibodies specific to eNOS (4μg; Type III, BD), ERβ (4μg; L-20, Santa Cruz) and ERβ (3μg, CWK-F12, DSHB). Negative controls were generated omitting antibody (NoAb). DNA fragments were recovered and analyzed by *q*RT-PCR as previously described [12]. Briefly, *q*RT-PCR were performed in duplicate or triplicate and the data, normalized to the corresponding DNA input control, were represented as Fold Induction +/-E2 or as Relative enrichment A.U. (Supplementary Figure 1).

Primers sequences for promoters, designed using Primer express 3.1 (Applied Biosystems), are as follows:

hNUP153 prom-eNOSpeak

5’-ACAATTTAACAACATCAACCATGTGA-3’ and 5’-CAAAAGTGTAGCAAAAGGATCTGATAA-3’.

### Statistical analysis

Data are expressed as mean ± SEM or as fold of induction as indicated in figures legend. Significance was calculated using a two-tailed *t*-test and/or one-way Analysis of Variance (ANOVA). *P* values of <0.05 were considered as significant in all tests.

## SUPPLEMENTARY MATERIALS AND FIGURES


