# Supplementary material for: Nucleoporin 153 regulates estrogen-dependent nuclear translocation of endothelial nitric oxide synthase and estrogen receptor beta in prostate cancer
Source: Oncotarget. 2018 Jun 15;9(46):27985–97. doi: 10.18632/oncotarget.25462 (PMC6021351; doi:10.18632/oncotarget.25462)
Supplement: Supplementary file 1 [file oncotarget-09-27985-s001.pdf]

Nucleoporin 153 regulates estrogen-dependent nuclear translocation of endothelial nitric oxide synthase and estrogen receptor beta in prostate cancer

SUPPLEMENTARY MATERIALS

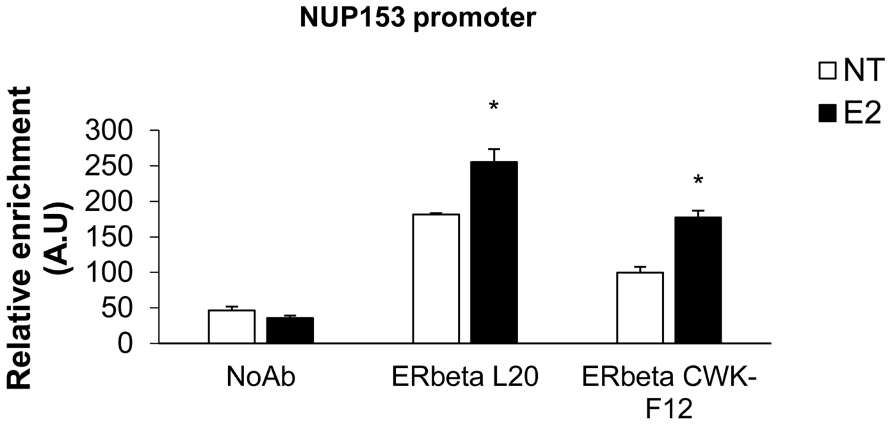

Supplementary Figure 1: ChIP analysis of Nup153 promoter with antibodies to ERβ L-20 (4µg), ERβ CWK-F12 (3µg) and no Antibody (NoAb), as control, in prostate cancer cells before and after estradiol treatment (NT and E_2, respectively). Data represent the mean +/-SEM. $^{*}p<0.05$ E_2 vs NT.

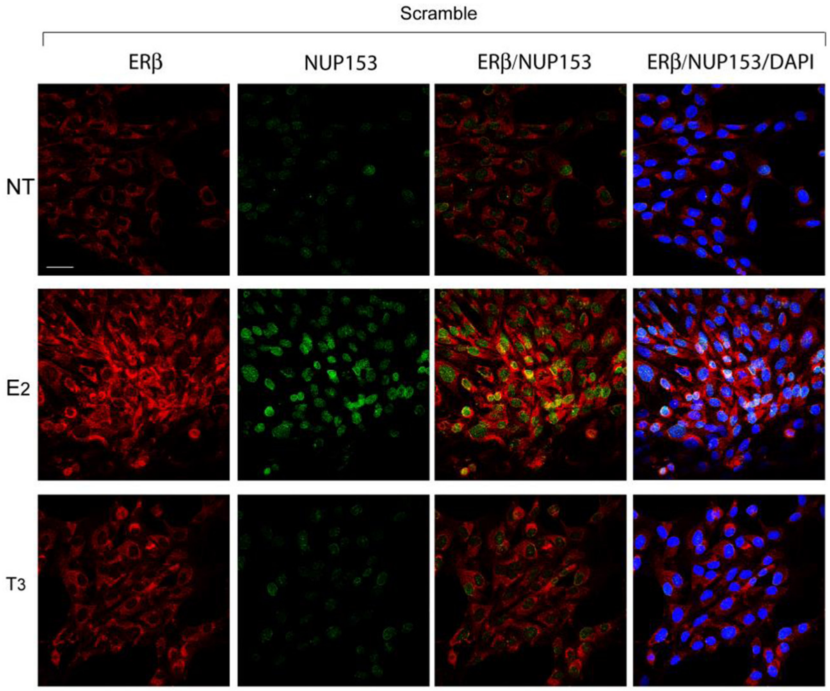

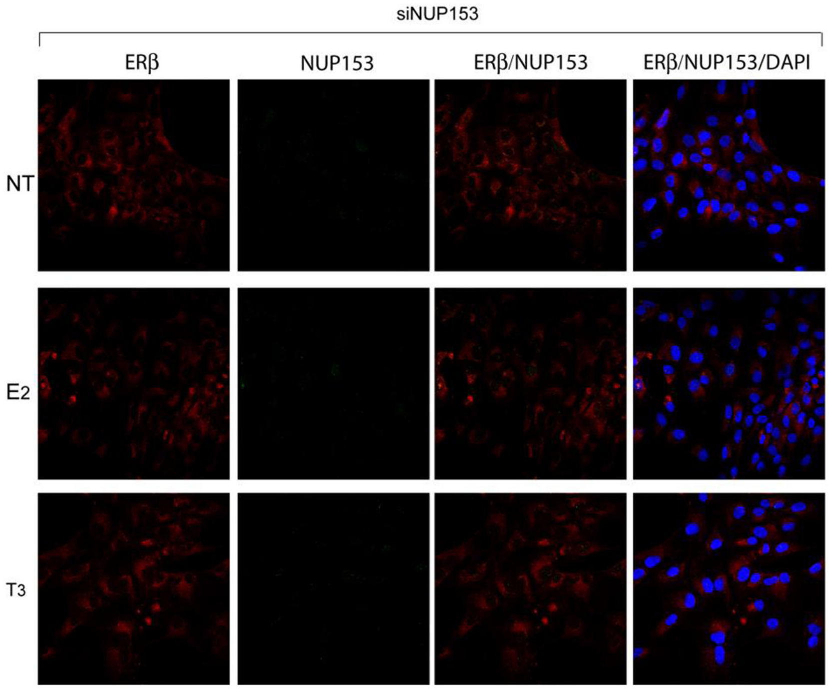

Supplementary Figure 2: Representative confocal microscopy images showing Nup153 (H-161; green) and ERβ (CWK-F12; red) expression in PCa cells before and after Nup153 depletion (lower panels), untreated or treated with E_2 (10^{-7}M, 3h 45min) or T_3 (10^{-7}M, 3h 45min). Cells silenced for Nup153 (60nM oligos mix) or treated with scramble control oligo were analysed at 48h post-transfection. T_3 treatment served as control of ERβ estrogen response specificity. Nuclei were counterstained with DAPI (blue).
